# Prognostic significance of sarcopenia in upper tract urothelial carcinoma patients treated with radical nephroureterectomy

**DOI:** 10.1002/cam4.795

**Published:** 2016-06-28

**Authors:** Hiroshi Fukushima, Yasukazu Nakanishi, Madoka Kataoka, Ken‐ichi Tobisu, Fumitaka Koga

**Affiliations:** ^1^ Department of UrologyTokyo Metropolitan Cancer and Infectious diseases Center Komagome Hospital3‐18‐22 Honkomagome Bunkyo‐kuTokyo113‐8677Japan

**Keywords:** Carcinoma, kidney pelvis, sarcopenia, survival, transitional cell, ureter

## Abstract

We investigated the prognostic significance of sarcopenia in upper tract urothelial carcinoma (UTUC) patients treated with radical nephroureterectomy (RNU). The skeletal muscle index (SMI) was calculated from computed tomography images. Sarcopenia was defined as SMI <43 cm^2^/m^2^ for males with body mass index <25 kg/m^2^, <53 cm^2^/m^2^ for males with BMI ≥25 kg/m^2^, and <41 cm^2^/m^2^ for females. Associations of sarcopenia with cancer‐specific survival (CSS) and overall survival (OS) were evaluated in 81 consecutive UTUC patients who underwent RNU. Forty‐seven (58%) out of 81 patients had sarcopenia. Multivariate analyses identified sarcopenia as a significant and independent poor prognostic factor for both CSS (hazard ratio [HR] 8.58, 95% confidence interval [CI]: 1.63–158.1, *P *=* *0.008) and OS (HR 6.05, 95%CI 2.00–26.21, *P *<* *0.001). In patients with locally advanced disease (pT3/4 or pN+), those with sarcopenia showed significantly worse CSS and OS than those without (5‐year CSS rate 55% vs. 100%, *P *=* *0.014; 5‐year OS rate 40% vs. 86%, *P *=* *0.007). In contrast, no prognostic difference was observed between patients with and without sarcopenia in those with organ‐confined disease (pTa‐2pN0/x). Sarcopenia is an independent poor prognostic factor for UTUC patients treated with RNU, particularly for those with locally advanced disease.

## Introduction

The incidence of upper urinary tract carcinoma (UTUC) remains low, but has increased to two cases per 100,000 person‐years in the United States 1. Although radical nephroureterectomy (RNU) is the standard of care for nonmetastatic UTUC patients, their prognosis after RNU is relatively poor; 20–30% develop metastatic recurrence and eventually die of the disease 2, 3. Thus, the identification of high‐risk patients as candidates for adjunctive therapies is essential in order to improve oncological outcomes. Previous studies investigated various prognostic factors in an attempt to accurately stratify the risk for mortality after RNU. Several pathological parameters, including the pT and pN stages, tumor grade, and lymphovascular invasion (LVI) have been established as prognostic factors in UTUC patients treated with RNU 4, 5. However, a relatively small number of studies have examined clinical parameters associated with the prognosis of these patients.

Sarcopenia, a novel concept reflecting the degenerative loss of skeletal muscle mass, develops as a critical pathophysiological change in the process of cancer cachexia 6, 7, 8. Sarcopenia has been associated with an unfavorable prognosis in patients with various cancers of the respiratory, gastrointestinal 7, 8, and urinary tracts 9, 10, 11, 12, 13. Regarding urological cancers, we recently reported the prognostic significance of sarcopenia in metastatic renal cell carcinoma patients 9 and advanced urothelial carcinoma patients for whom curative surgery was not indicated 10. The prognostic impact of sarcopenia was also demonstrated in bladder cancer patients treated with radical cystectomy 12. In this study, we investigated the prognostic role of sarcopenia in operable UTUC patients treated with RNU.

## Materials and Methods

### Patients

This study protocol (approval number 1420) was approved by the Institutional Ethical Committee. This retrospective study consisted of 85 consecutive Japanese UTUC patients who underwent RNU at a single cancer center between November 2001 and February 2015. Four patients were excluded (one due to the lack of pathological findings and three due to less than 3 months follow‐up) and the remaining 81 patients were subjects for analysis. All patients underwent open or gasless retroperitoneoscopic RNU 14 for nonmetastatic UTUC. A bladder cuff was excised by the extravesical approach. The regional lymph nodes were dissected in patients with swollen nodes detected radiologically or intraoperatively. All patients were preoperatively assessed by computed tomography (CT) within 1 month prior to RNU. Variables included age at RNU, gender, body mass index (BMI), Eastern Cooperative Oncology Group performance status (ECOG PS), tumor location, preoperative C‐reactive protein (CRP), tumor grade, pT and pN stages, LVI, and neoadjuvant and/or adjuvant therapy. BMI was calculated as follows: BMI (kg/m^2^) = ((weight)/(height)^2^). BMI was categorized using a cut‐off value of 25 kg/m^2^ according to the WHO criteria of overweight/obesity specific for Asia‐Pacific populations 15. The cut‐off point of CRP was determined according to a previous study 16. Tumors were staged and graded in accordance with the 2002 TNM classification and 1973 WHO grading system, respectively. LVI was defined as the existence of tumor cells within an endothelium‐lined space with no underlying muscular walls.

### Image analysis

Computed Tomograpy images taken within 1 month prior to RNU were analyzed as described previously 9, 10. Briefly, the third lumbar vertebra (L3) was set as a landmark, and the cross‐sectional areas of skeletal muscle were measured in two consecutive slices of axial CT images using Hounsfield unit thresholds of −29 to +150. The mean value was calculated for each patient and normalized for stature: skeletal muscle index (SMI) (cm^2^/m^2^) = ((skeletal muscle cross‐sectional area at L3)/(height)^2^). SMI was considered as an indicator of whole‐body muscle mass because the total lumbar‐skeletal muscle cross‐sectional area linearly correlates with whole‐body muscle mass 17. OsiriX Imaging Software (Pixmeo, Geneva, Switzerland) was used for analyses. All image analyses were performed by one investigator (H.F.) who was blinded to other clinicopathological parameters and outcomes.

The cut‐off values of SMI to best discriminate cancer‐specific survival (CSS) were explored for the definition of sarcopenia as described previously 9, 10. Among the various cut‐off values reported, a univariate Cox proportional model yielded the highest hazard ratio (HR) (9.18, *P *=* *0.005) when the cut‐off values of SMI incorporating BMI, proposed by Martin et al. 7, were utilized. Thus, sarcopenia was defined as SMI <43 cm^2^/m^2^ for males with BMI <25 kg/m^2^, <53 cm^2^/m^2^ for males with BMI ≥25 kg/m^2^, and <41 cm^2^/m^2^ for females in this study 7.

### Statistical analysis

Differences in the distribution of variables between groups were evaluated using the chi‐squared test for categorical variables and Wilcoxon rank‐sum test for continuous variables. CSS was calculated from the date of RNU to death due to UTUC or the last follow‐up. overall survival (OS) was calculated from the date of RNU to death or the last follow‐up. Survival curves were generated using the Kaplan–Meier method and compared using the log‐rank test. The Cox proportional hazards model was used to evaluate the relationships between variables and survival. Variables with *P *<* *0.10 in the univariate analysis were included in the multivariate analysis. A reduced multivariate model was developed using the stepwise backward method, in which the variable with the highest *P*‐value was eliminated from each iteration of the multivariate analysis. All statistical analyses were performed using JMP 9.0.2 (SAS Institute Inc., Cary, NC). Significance required two‐tailed *P *<* *0.05.

## Results

### Patient and tumor characteristics

The demographical data of patients and tumors are listed in Table 1. The median (range) age at RNU was 71 (41–87) years. The kidney, ureter, and both were involved in 36 (44%), 31 (38%), and 14 patients (17%), respectively. Seven patients (9%) received neoadjuvant therapy: platinum‐based chemotherapy (four patients) and chemoradiotherapy (three patients). Fifteen patients (19%) were treated with adjuvant therapy: platinum‐based chemotherapy (13 patients), gemcitabine monotherapy (one patient), and chemoradiotherapy (one patient). Twenty‐two (27%), 9 (11%), 6 (7%), 38 (47%), and 6 patients (7%) had pTa/is, pT1, pT2, pT3, and pT4 tumors, respectively. Regional lymph nodes were dissected in 29 patients (36%) and seven (24%) had positive nodes. Median (range) SMI (cm^2^/m^2^) were 44.5 (29.4–60.2) and 38.1 (29.6–50.2) for males and females, respectively. Forty‐seven patients (58%) were diagnosed with sarcopenia.

**Table 1:** Patient and tumor characteristics and their relationships with sarcopenia

Variables		Total, *n* (%)	Sarcopenia, *n* (%)		*P*‐value
			Yes	No	
No. of patients		81 (100)	47 (58)	34 (42)	
Age at RNU (years), median (range)		71 (41–87)	71 (55–85)	70 (41–87)	0.55
Gender	Male	53 (65)	28 (60)	25 (74)	0.19
	Female	28 (35)	19 (40)	9 (26)	
ECOG PS	0	61 (75)	35 (74)	26 (76)	0.84
	1≤	20 (25)	12 (26)	8 (24)	
BMI	<25 kg/m^2^	63 (78)	36 (77)	27 (79)	0.76
	≥25 kg/m^2^	18 (22)	11 (23)	7 (21)	
SMI (cm^2^/ m^2^), median (range)	Male	44.5 (29.4–60.2)	39.1 (29.4–50.8)	48.5 (43.2–60.2)	<0.001
	Female	38.1 (29.6–50.2)	35.6 (29.6–40.1)	45.0 (41.9–50.2)	<0.001
Tumor location	Kidney	36 (44)	20 (42)	16 (47)	0.89
	Ureter	31 (38)	19 (40)	12 (35)	
	Both	14 (17)	8 (17)	6 (18)	
Tumor grade	G1/2	31 (38)	16 (34)	15 (44)	0.36
	G3	50 (62)	31 (66)	19 (56)	
pT stage	pTa‐2	37 (46)	17 (36)	20 (59)	0.043
	pT3/4	44 (54)	30 (64)	14 (41)	
pN stage	pN0	22 (27)	11 (23)	11 (32)	0.56
	pNx	52 (64)	31 (66)	21 (62)	
	pN+	7 (9)	5 (11)	2 (6)	
LVI	No	50 (62)	25 (53)	25 (74)	0.063
	Yes	31 (38)	22 (47)	9 (26)	
Neoadjuvant therapy	No	74 (91)	41 (87)	33 (97)	0.12
	Yes	7 (9)	6 (13)	1 (3)	
Adjuvant therapy	No	66 (81)	38 (81)	28 (82)	0.86
	Yes	15 (19)	9 (19)	6 (18)	
CRP	<5 mg/L	59 (73)	32 (68)	27 (79)	0.26
	≥5 mg/L	22 (27)	15 (32)	7 (21)	

RNU, radical nephroureterectomy; ECOG PS, Eastern Cooperative Oncology Group performance status; BMI, body mass index; SMI, skeletal muscle index; LVI, lymphovascular invasion; CRP, C‐reactive protein.

### Relationships between sarcopenia and clinicopathological variables

Relationships between sarcopenia and clinicopathological variables are also shown in Table 1. Sarcopenia was associated with higher pT stage significantly (*P *=* *0.043) and positive LVI with marginal significance (*P *=* *0.063) but not with ECOG PS (*P *=* *0. 84) or CRP (*P *=* *0.26).

### Impact of sarcopenia on survival

**Figure 1:**
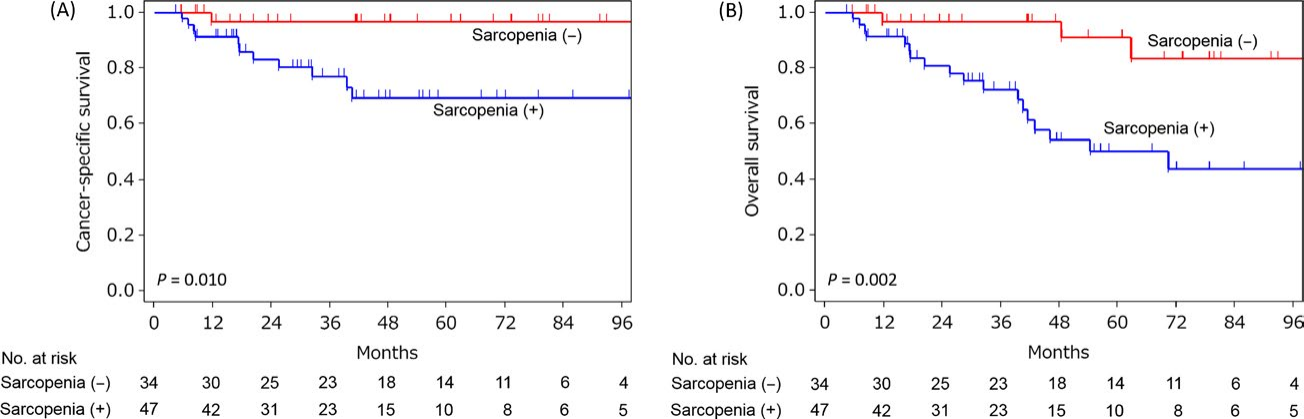
Cancer‐specific survival (A) and overall survival (B) curves according to the presence or absence of sarcopenia.

During the follow‐up (median 41 months, range 4–170), 21 patients died of any causes and 12 died of UTUC. The 5‐year CSS and OS rates for the whole cohort were 81% and 67%, respectively. Patients with sarcopenia showed significantly worse CSS (5‐year CSS rate 69% vs. 97%, *P *=* *0.010; Fig. 1A) and OS (5‐year OS rate 50% vs. 91%, *P *=* *0.002; Fig. 1B) than those without. In the univariate analysis, tumor grade, pT and pN stages, LVI, neoadjuvant therapy, adjuvant therapy, and sarcopenia were significantly associated with CSS (Table 2). The multivariate analysis identified sarcopenia as a significant and independent poor prognostic factor for both CSS (HR 8.58, *P *=* *0.008) and OS (HR 6.05, *P *<* *0.001) along with pN+ disease for both CSS (HR 8.45, *P *=* *0.004) and OS (HR 5.62, *P *=* *0.012; Table 2).

**Table 2:** Univariate and multivariate analyses for predicting cancer‐specific and overall survival

Variables		Cancer‐specific survival						Overall survival					
		Univariate			Multivariate			Univariate			Multivariate		
		HR	95% CI	*P*‐value	HR	95% CI	*P*‐value	HR	95% CI	*P‐*value	HR	95% CI	*P*‐value
Age at RNU (years)		0.99	0.94–1.06	0.81				1.01	0.96–1.06	0.74			
Gender	Male	ref.		0.83				ref.		0.60			
	Female	0.88	0.23–2.80					0.78	0.30–1.89				
ECOG PS	0	ref.		0.97				ref.		0.38			
	1≤	0.98	0.22–3.28					1.51	0.57–3.64				
BMI	<25 kg/cm^2^	ref.		0.95				ref.		0.54			
	≥25 kg/cm^2^	1.04	0.23–3.50					0.72	0.21–1.95				
Tumor location	Kidney	ref.		0.92				ref.		0.85			
	Ureter	1.25	0.35–4.51					0.93	0.36–2.29				
	Both	1.30	0.19–6.08					0.66	0.10–2.45				
Tumor grade	G1/2	ref.		0.042				ref.		0.12			
	G3	3.99	1.05–26.03					2.07	0.84–5.82				
pT stage	pTa‐2	ref.		0.003				ref.		0.005			
	pT3/4	9.92	1.93–181.4					4.04	1.49–14.06				
pN stage	pN0/x	ref.		0.003	ref.		0.004	ref.		0.019	ref.		0.012
	pN+	9.08	2.38–29.65		8.45	2.12–30.42		4.75	1.35–13.05		5.62	1.53–17.00	
LVI	No	ref.		0.020				ref.		0.038			
	Yes	3.95	1.24–14.87					2.51	1.05–6.08				
Neoadjuvant therapy	No	ref.		0.032				ref.		0.023			
	Yes	5.38	1.19–18.25					4.46	1.27–12.21				
Adjuvant therapy	No	ref.		0.025				ref.		0.18			
	Yes	4.14	1.22–13.06					2.10	0.68–5.42				
CRP	<5 mg/L	ref.		0.63				ref.		0.20			
	≥5 mg/L	1.35	0.36–4.29					1.82	0.72–4.34				
Sarcopenia	No	ref.		0.005	ref.		0.008	ref.		0.001	ref.		<0.001
	Yes	9.18	1.78–167.9		8.58	1.63–158.1		5.59	1.88–23.91		6.05	2.00–26.21	

HR, hazard ratio; CI, confidence interval; RNU, radical nephroureterectomy; ECOG PS, Eastern Cooperative Oncology Group performance status; BMI, body mass index; LVI, lymphovascular invasion; CRP, C‐reactive protein; ref, reference.

**Figure 2:**
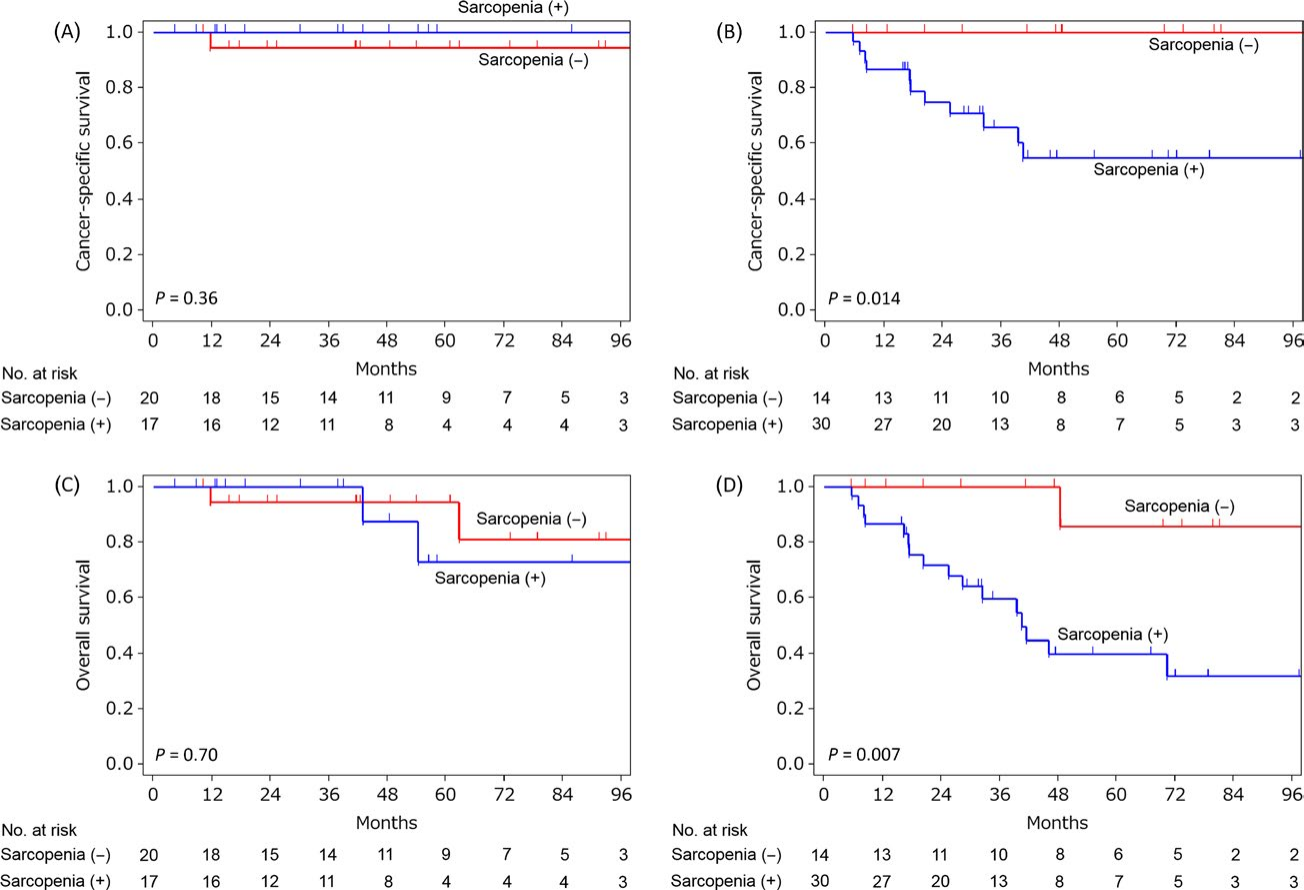
Cancer‐specific (A and B) and overall survival curves (C and D) according to the presence or absence of sarcopenia in patients with organ‐confined disease (pTa‐2pN0/x, A and C) and locally advanced disease (pT3/4 or pN+, B and D).

We further investigated the prognostic value of sarcopenia in the subgroups: organ‐confined (pTa‐2pN0/x) and locally advanced disease (pT3/4 or pN+). In patients with organ‐confined disease, neither CSS nor OS was significantly different between those with and without sarcopenia (5‐year CSS rate 100% vs. 94%, Fig. 2A; 5‐year OS rate 73% vs. 94%, Fig. 2C). In contrast, significant differences were observed in patients with locally advanced disease; 5‐year CSS and OS rates for patients with and without sarcopenia were 55% versus 100% (*P *=* *0.014; Fig. 2B) and 40% versus 86% (*P *=* *0.007; Fig. 2D), respectively.

## Discussion

This study is the first to demonstrate the prognostic significance of sarcopenia in UTUC patients treated with RNU. Sarcopenia was a strong and independent prognostic factor among the various clinicopathological parameters examined. The prognostic significance of sarcopenia was evident in patients with locally advanced disease (pT3/4 or pN+) but not in those with organ‐confined disease (pTa‐2pN0/x). This result is conceivable considering that sarcopenia has been identified as an unfavorable prognostic factor in advanced urothelial carcinoma patients 10.

However, the reason why sarcopenia has a prognostic impact only for locally advanced disease is currently unclear. The development of sarcopenia involves both tumor and host factors. Indeed, sarcopenia was observed more frequently in patients with locally advanced disease than those with organ‐confined disease (68% vs. 46%, *P *=* *0.043). Our results suggest that, as a tumor factor, locally advanced disease that induces sarcopenia is aggressive; and, as a host factor, patients with sarcopenia are prone to or those without are unlikely to die from locally advanced UTUC. Sarcopenia was not a prognostic factor for organ‐confined disease presumably because of its less aggressive nature.

Sarcopenia clearly stratified the prognosis of locally advanced UTUC patients treated with RNU. Patients without sarcopenia appear to show favorable CSS even though they have locally advanced disease. In contrast, the prognosis of sarcopenic patients with locally advanced disease was extremely poor. These patients might be candidates for adjuvant therapies. However, cautions should be paid because patients with sarcopenia are reportedly susceptible to the adverse events of anticancer therapies 18. Thus, sarcopenia may be helpful in counseling patients on their personalized risk to determine therapeutic strategies including adjuvant therapies and clinical trials.

Cancer cachexia, characterized by fatigue, loss of appetite, weight loss, and frailty, involves host inflammatory responses to tumors 6, 19, 20, 21. Several studies have demonstrated the prognostic roles of cancer cachexia‐related factors in UTUC patients treated with RNU. CRP, a nonspecific systemic inflammatory marker, has been identified as a useful biomarker for predicting a poor prognosis in UTUC patients treated with RNU 16, 22. However, the clinical usefulness of CRP as a cancer biomarker is limited by its nonspecific elevation due to noncancer‐related conditions including infection, autoimmune disease, and cardiovascular disease. This study did not detect a significant relationship between CRP and survival, possibly because we did not exclude patients with nonspecific CRP elevations. Based on the prognostic significance of sarcopenia beyond CRP demonstrated in this study and the noncancer‐specific nature of CRP, sarcopenia appears to surpass CRP as a prognostic factor associated with host inflammatory responses to tumors in UTUC patients treated with RNU.

Body weight and BMI decrease with the development of cancer cachexia. In this study, BMI was not significantly associated with CSS and OS. Similarly, the prognostic roles of decreased BMI in UTUC patients have been inconclusive in previous studies 23, 24. This may partly be because BMI does not reflect body composition, including the proportion of fat to muscle tissue and the degree of fluid accumulation such as ascites and edema 8. Moreover, the contemporary trend of increasing obesity obscures the clinical significance of weight loss 7.

Sarcopenia has several strengths in its clinical use, with the most advantageous being that it is easy and simple to evaluate with no additional cost. CT scanning, which is universally available in clinical practice, is required for the diagnosis, staging, and follow‐up of UTUC patients. The calculation of SMI is easy and simple using free software and CT images without special training. Furthermore, sarcopenia is a highly objective measure because it is possible to estimate the composition of the human body using CT scans with a reported precision error of 1.4% 25. Sarcopenia is also stable; rapid fluctuations in skeletal muscle volumes in a short interval are unlikely to occur. The objectivity and stability of sarcopenia appears to be advantageous for correctly and precisely predicting patient prognoses.

This study has several limitations. It is limited by its inherent retrospective nature and relatively small patient cohort at a single institution. Thus, our results need to be validated in larger multicenter cohorts. In this study, established prognostic factors including tumor grade, pT stage, and LVI, were not identified as independent prognostic factors in multivariate analysis probably because of a small sample size. In addition, a significant association between pT stage and sarcopenia attenuated the prognostic significance of pT stage in multivariate analysis including sarcopenia. Indeed, pT stage was an independent predictor for both CSS and OS when excluding sarcopenia from multivariate analysis (data not shown). Furthermore, our cohort is heterogeneous in terms of RNU‐related adjunctive therapies. However, sarcopenia was still a significant predictor of worse CSS and OS when analyzed in patients who did not receive neoadjuvant therapy (data not shown). Regional lymph node dissection was not performed in most patients. In survival analyses, patients with pNx and pN0 were grouped together because CSS and OS curves were similar between those with pNx and pN0. In addition, weight loss, one of the symptoms of cancer cachexia, and systemic inflammation‐related parameters (e.g., the neutrophil‐to‐lymphocyte ratio, fibrinogen, and interleukin‐6) were not evaluated in this study. Moreover, the BMI‐incorporated cut‐off values of SMI based on a large Canadian patient cohort were used to define sarcopenia in this study 7. Since these cut‐off values have not been validated in a Japanese population, future studies may find more appropriate definitions of sarcopenia for Japanese patients. However, the prognostic significance of sarcopenia has been demonstrated in Japanese patients with various urological cancers using BMI‐incorporated cut‐off values 9, 10, indicating that this definition is optimal. Finally, we did not investigate the role of salvage chemotherapy in the management of sarcopenic UTUC patients developing local and/or distant recurrence after RNU. Sarcopenic patients may be unlikely to receive salvage chemotherapy at adequate doses due to poor performance status and severe adverse events, which might lead to worse clinical outcomes. Future studies are required to evaluate this point.

## Conclusions

We demonstrated for the first time that sarcopenia is a strong and independent prognostic factor in UTUC patients treated with RNU. Sarcopenia clearly stratified the prognosis of patients with locally advanced disease. Thus, sarcopenia may be helpful in counseling patients on their personalized risk to determine therapeutic strategies. Our preliminary results warrant validation in large multicenter studies.

## Conflict of Interest

None of the authors have financial or other relationships that would constitute a conflict of interest.
